# 9.4 COMPLEX SYSTEM THEORY AND THE TRANSDIAGNOSTIC USE OF EARLY WARNING SIGNALS TO FORESEE THE TYPE OF FUTURE TRANSITIONS IN SYMPTOMS

**DOI:** 10.1093/schbul/sby014.032

**Published:** 2018-04-01

**Authors:** Marieke Wichers, Marieke Schreuder, Catharina Hartman, Hanneke Wigman

**Affiliations:** Interdisciplinary Center Psychopathology and Emotion regulation (ICPE), University Medical Center Groningen

## Abstract

**Background:**

Recently, we showed that assumptions from complex system theory seem applicable in the field of psychiatry. This means that indicators of critical slowing down in the system signal the risk for a critical transition in the near future. In the current study we wanted to explore whether the principle of critical slowing down may also be informative to anticipate on the type of symptoms that individuals are most likely to develop. This is relevant as it may lead to personalized prediction of risk of whether adolescents with mixed complaints are most likely to develop either depression, anxiety, somatic or psychotic symptoms in the near future. For example, we hypothesized that critical slowing down in feeling ‘suspicious’ more strongly indicates risk for a future transition to psychotic symptoms, while critical slowing down in feeling ‘down’ more strongly indicates risk for a transition to depressive symptoms.

**Methods:**

We examined this in a population of adolescents (most between 15 and 18 years) as adolescents are an at-risk group for the development of psychopathology. At baseline experience sampling was performed for 6 days, 10 measurements a day. Affect items were used to assess autocorrelation as an indicator of ‘critical slowing down’ of the system. At baseline and follow-up SCL-90 questionnaires were administered. In total, 147 adolescents participated both in baseline and follow-up measures and showed increases in at least one of the defined symptom dimensions. We examined whether autocorrelation was positively associated with the size of symptom transition and whether different type of transitions (in depression, anxiety etc.) were differentially predicted by autocorrelations in specific affect states.

**Results:**

The analyses were done very recently, and findings have not been presented before. We found both shared and specific indicators of risk in the development for transition to various symptom dimensions. First, autocorrelation in ‘feeling suspicious’ appeared to be the strongest signal for all assessed psychopathology dimensions (SCL-90 depression: std beta: 0.185; p <0.001; SCL-90 anxiety: std beta: 0.093; p=0.006; SCL-90 interpersonal sensitivity: std beta: 0.176, p<0.001). Second, we found that the combination of ‘feeling suspicious’ and the affect with the second-highest autocorrelation together predicted the precise type of symptom transition. Thus, the combination of feeling suspicious (std beta: 0.185; p<0.001) and down (std beta: 0.108; p=0.001) predicted larger increases in depressive symptoms one year later on the SCL-90, while the combination of feeling suspicious (std beta: 0.093; p=0.006) with feeling anxious (std beta: 0.086; p=0.014) predicted larger increases in anxiety symptoms a year later on the SCL-90.

**Discussion:**

These findings support the hypothesis that indicators of slowing down can not only be used to predict risk for a mean level shift in symptoms, but that they can also be informative for the type of symptom transitions at hand. In a next step these findings could be translated to designs measuring personalized early warnings for future direction of symptom shifts, and if successful to clinical implementation of these techniques.

